# Learning to shape virtual patient locomotor patterns: internal representations adapt to exploit interactive dynamics

**DOI:** 10.1152/jn.00408.2018

**Published:** 2018-11-07

**Authors:** Christopher J. Hasson, Sarah E. Goodman

**Affiliations:** ^1^Neuromotor Systems Laboratory, Department of Physical Therapy, Movement, and Rehabilitation Sciences, Northeastern University, Boston, Massachusetts; ^2^Department of Bioengineering, Northeastern University, Boston, Massachusetts; ^3^Department of Biology, Northeastern University, Boston, Massachusetts

**Keywords:** biomechanics, internal model, locomotion, motor learning, rehabilitation, stroke

## Abstract

This work aimed to understand the sensorimotor processes used by humans when learning how to manipulate a virtual model of locomotor dynamics. Prior research shows that when interacting with novel dynamics humans develop internal models that map neural commands to limb motion and vice versa. Whether this can be extrapolated to locomotor rehabilitation, a continuous and rhythmic activity that involves dynamically complex interactions, is unknown. In this case, humans could default to model-free strategies. These competing hypotheses were tested with a novel interactive locomotor simulator that reproduced the dynamics of hemiparetic gait. A group of 16 healthy subjects practiced using a small robotic manipulandum to alter the gait of a virtual patient (VP) that had an asymmetric locomotor pattern modeled after stroke survivors. The point of interaction was the ankle of the VP’s affected leg, and the goal was to make the VP’s gait symmetric. Internal model formation was probed with unexpected force channels and null force fields. Generalization was assessed by changing the target locomotor pattern and comparing outcomes with a second group of 10 naive subjects who did not practice the initial symmetric target pattern. Results supported the internal model hypothesis with aftereffects and generalization of manipulation skill. Internal models demonstrated refinements that capitalized on the natural pendular dynamics of human locomotion. This work shows that despite the complex interactive dynamics involved in shaping locomotor patterns, humans nevertheless develop and use internal models that are refined with experience.

**NEW & NOTEWORTHY** This study aimed to understand how humans manipulate the physics of locomotion, a common task for physical therapists during locomotor rehabilitation. To achieve this aim, a novel locomotor simulator was developed that allowed participants to feel like they were manipulating the leg of a miniature virtual stroke survivor walking on a treadmill. As participants practiced improving the simulated patient’s gait, they developed generalizable internal models that capitalized on the natural pendular dynamics of locomotion.

## INTRODUCTION

Locomotor impairments are a significant contributor to disability, comorbidities, diminished self-care, and loss of self-reliance, and their prevalence is increasing in the global aging population (Satariano et al. 2012). Rehabilitation is typically prescribed to restore a more functional gait pattern and is traditionally performed by human therapists or, more recently, with robotic exoskeletons. Although rehabilitation outcomes are multifactorial and often positive, a significant percentage of patients retain locomotor impairments after locomotor training (e.g., see reviews by Belda-Lois et al. 2011; Díaz et al. 2011; Louie et al. 2015; Nam et al. 2017; Pennycott et al. 2012; Schaechter 2004).

To improve the effectiveness of locomotor rehabilitation, studies have focused on understanding patient learning processes. Various approaches have been tested that address elements such as the presentation of error feedback, repetition of movement, training specificity, and focus of attention (Patton et al. 2006; Richards et al. 1993; Yogev‐Seligmann et al. 2008). However, the complementary question remains largely unanswered: how do therapists learn to manipulate patient locomotor patterns? This question can be reframed in terms of motor adaptation: how does a human learn to interact with and manipulate the moving leg of a patient, while at the same time compensating for the high dynamical complexity afforded by linked-segmental dynamics under external and possibly pathological neuromuscular control?

To answer this question, one can look toward current theories of motor adaptation based on evidence that humans develop and use internal models of externally imposed dynamics. Internal models are neural representations of dynamics that allow humans to map neural commands to limb motion, and vice versa, providing the ability to predict and generalize future limb states (Kawato 1999). Evidence for internal model use by humans has been provided by the use of externally applied force fields of varying complexity. This includes the classic velocity-dependent curl field (Kurtzer et al. 2008; Lackner and Dizio 1994; Scheidt et al. 2001; Shadmehr and Mussa-Ivaldi 1994; Thoroughman and Shadmehr 2000) and others that include combined gravitoinertial and Coriolis fields (Lackner and Dizio 1998), negative viscosity (Huang et al. 2010), inverted pendulums (Mah and Mussa-Ivaldi 2003), mass-spring dynamics (Dingwell et al. 2002), and hammerlike objects (Ingram et al. 2010).

Although the body of research on internal models is robust, it remains unclear whether the internal model hypothesis holds ad infinitum, i.e., when controlling increasingly complex dynamical systems, such as the leg of a locomoting patient, humans could resort to model-free strategies (Haith and Krakauer 2013; Huang et al. 2011) such as a stiffness strategy with high antagonistic coactivation (Howard et al. 2011) or a rote memorization strategy (Conditt et al. 1997; Conditt and Mussa-Ivaldi 1999). Examples of model-free learning have been shown in several task domains. For instance, despite years of driving experience, humans perform incorrect steering operations when asked to perform a simple lane shift without visual feedback, which suggests a reliance on model-free feedback control (Wallis et al. 2002).

Improving our understanding of human adaptation to complex dynamics could lead to future improvements in human- and robot-delivered locomotor rehabilitation. For example, there is no specific set of standardized instructions detailing how a therapist is to help patients achieve locomotor goals with manual assistance, and quantitative measurements of human therapists show large differences in manipulative actions between therapists for the same patient (Galvez et al. 2005, 2007). A deeper understanding of therapist adaptation may also advance robotic gait training algorithms by incorporating beneficial aspects of human-delivered approaches (biomimicry), such as adaptive impedance control (Hussain et al. 2013) and assistance-as-needed (Cai et al. 2006).

Whether and how humans develop internal models of locomotor dynamics is unknown, in part because of the challenge of conducting controlled studies with real therapists and patients, as both adapt simultaneously. This study overcomes this challenge by asking subjects to interact with a locomoting virtual patient (VP) with the use of a robotic manipulandum. The VP’s locomotor dynamics are mathematically specified with a simple impedance model that aims to capture the basic features of human locomotion. Internal model formation is probed with a classical force-field approach with a locomotor twist: the field consists of pathological gait dynamics using chronic stroke as a model. Interaction with the VP causes subjects to experience forces that pull their hands into a typical stroke gait pattern as if they were holding onto the leg of a real (miniaturized) patient. Subjects must learn how to apply forces to the VP’s leg to make its gait pattern symmetric. The overall hypothesis is that subjects learn internal models of external locomotor dynamics, which would be supported by the presence of aftereffects (i.e., persistence of feedforward motor plans despite altered dynamics) in response to randomly interspersed catch trials (H1) and generalization of manipulation skill to a different locomotor training task (H2). The alternative hypothesis is that subjects use a purely model-free manipulation strategy (e.g., high arm stiffness or rote memorization).

## METHODS

### Locomotor Simulator

#### Stroke locomotor kinematics model.

The VP was modeled after the locomotor patterns of stroke survivors, characterized by less weight bearing and shorter steps in the affected leg (Roth et al. 1997). The model was based on existing published data presented in Olney and Richards (1996). The aim was to create a model that had enough detail to approximate pathological locomotor dynamics yet remained mathematically tractable to implement in a real-time physically interactive simulation. Joint angles were obtained through manual digitization of hip and knee joint angle data (θ_h_ and θ_k_, respectively) in the sagittal plane throughout the gait cycle for the medium self-selected walking speed (0.41 m/s) hemiparetic patient group in Olney and Richards (1996) (their Fig. 2b). The digitized data were fit with piecewise cubic splines using a linear least-squares solver with the constraint that the *y*-value and slope had to be equal in the beginning (0%) and end (100%) of the gait cycle. Although the Olney and Richards data were normalized to the stride cycle, the stride time was needed to determine where in the gait cycle the VP is during simulation. With the reported average walking velocity of 0.41 m/s the stride frequency was estimated to be 0.64 strides/s, giving a stride time of 1.56 s based on Wagenaar and Beek (1992). In the experiment, subjects could see the motion of both the unaffected and affected legs of the VP but could only physically interact with the affected limb (see *Experimental Approach*). 

#### Kinematic targets for adaptation experiment.

**Fig. 1.**
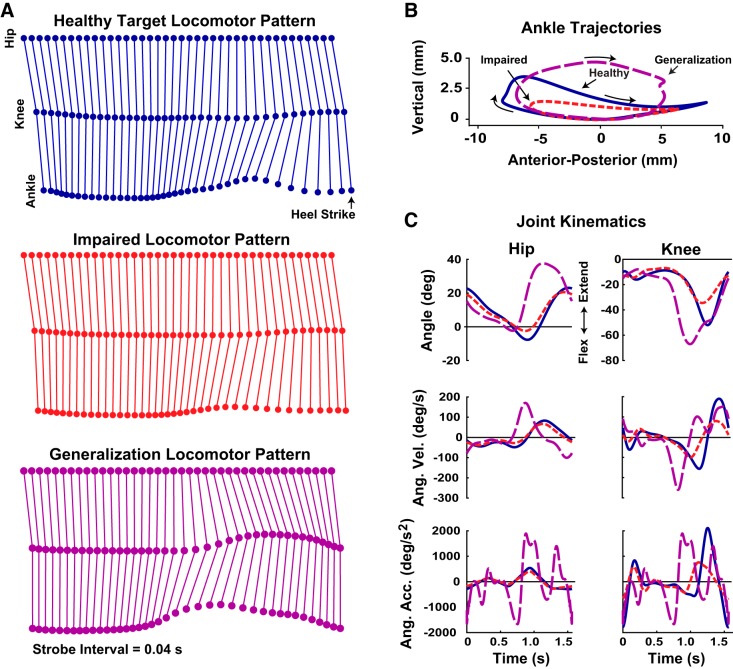
Kinematics used to create the virtual patient (VP) model. *A*: stick figure representations of the VP kinematics. The nominal impaired locomotor pattern (red) shows how the VP’s affected leg tried to move on its own, absent input from the subjects. The task for subjects was to make the VP move in the healthy target pattern (blue). The stroke and healthy target patterns were derived from real stroke patient data (Olney and Richards 1996) based on motions of the affected and unaffected legs, respectively. The generalization locomotor pattern (purple) was based on new experimental data collected for the study. *B*: 2-dimensional VP ankle trajectory for each condition. *C*: hip and knee VP angular kinematics for each condition as a function of time.

For some experimental conditions subjects were asked to make the VP’s gait symmetric, i.e., make the affected leg follow the same kinematic pattern as the unaffected leg. This “healthy” target trajectory was created by digitizing the contralateral leg kinematics for the stroke survivor data presented in Olney and Richards and shifting the phase by 180° (Fig. 1*A*). Here, we use the term “healthy” in a relative sense, i.e., in stroke survivors the unaffected leg typically exhibits a kinematic pattern closer to healthy adults compared with the affected leg. The healthy target trajectory was shown to subjects, and the task was to follow this target trajectory with appropriate timing (see *Experimental Approach* for more details). To test generalization, subjects were asked to make the VP’s ankle follow a different trajectory, which was created by measuring the gait kinematics of a healthy male adult executing a rendition of the “not particularly silly” walk performed by Michael Palin in “The Ministry of Silly Walks” sketch from the *Monty Python’s Flying Circus* sketch comedy show (episode 14). This walk has an exaggerated step height with a hesitation at the end (Fig. 1*B*). For this, a 12-camera passive reflective marker motion tracking system was used, sampling at 120 Hz (OptiTrack Flex 13; NaturalPoint, Corvallis, OR), with markers placed on the hip, knee, and right lateral malleolus. After data collection, a single representative gait cycle was selected, and the data were resampled to match the stroke stride time (1.56 s; Fig. 1*C*).

#### Robotic interface.

A small robotic manipulandum (Geomagic Touch; 3D Systems, Andover, MA) was used to haptically render the VP’s locomotor dynamics. The manipulandum allows six-degree of freedom movement and renders three-dimensional forces at the end-effector (position resolution: 0.055 mm; backdrive friction: 0.26 N; max force output: 3.3 N; stiffness: 1.26, 2.31, and 1.02 N/mm for *X*, *Y*, and *Z* directions) within a small workspace (160 mm W × 120 mm H × 70 mm D). The device used an open-loop impedance control scheme (Hatzfeld and Kern 2016), i.e., it measured its position and output a programmed force upon the user. The manipulandum end-effector defined a single point at which the user interacted with the VP. This point was the ankle joint (lateral malleolus) of the VP’s affected leg, which is close to the points of lower-extremity force application commonly used in body weight-supported gait rehabilitation (Kosak and Reding 2000). The rendered motion of the VP was scaled down by ~96.5% (1.0 m in real-patient dimensions = 0.035 m in manipulandum workspace) so that it required only small wrist/forearm motions. The VP model was two dimensional, and manipulandum end-effector motion was restricted to the frontal plane of the subject.

#### Rigid body dynamics model.

The VP’s affected leg was modeled with the equations of motion for a two-dimensional double pendulum (thigh and shank) derived by the Lagrangian method (Bruderlin and Calvert 1989; Sasagawa et al. 2014; van der Kooij et al. 1999). For simplicity, the hip joint was considered to be fixed (upper body dynamics were excluded) and the foot segment was omitted. Rigid body dynamics were defined by inertial, centrifugal, and gravitational torques,

(1) $$I\left(\theta \right)\overset{¨}{\theta }=T+V\left(\theta ,\overset{˙}{\theta }\right)+G\left(\theta \right)$$

where **I**(**θ**) is the geometry-dependent inertia tensor, $V\left(\theta ,\overset{˙}{\theta }\right)$ are the torques due to centrifugal and Coriolis forces, and **G**(**θ**) are the gravitational torques. In *Eq. 1*, **T** = [T_h_,T_k_]^T^ (vector of joint torques) and the quantities ***θ*** = [θ_h_,θ_k_]^T^, $\dot{\theta }={\left[{\dot{\theta }}_{\text{h}},{\dot{\theta }}_{\text{k}}\right]}^{\text{T}}$, and $\overset{¨}{\theta }={\left[{\overset{¨}{\theta }}_{\text{h}},{\overset{¨}{\theta }}_{\text{k}}\right]}^{\text{T}}$ are vectors of joint angles, velocities, and accelerations, respectively. The subscripts h and k represent the hip and knee, respectively (superscript T denotes transposition). The **I**, **V**, and **G** matrices are detailed in the appendix. Values for segment lengths, masses, and inertias are given in the appendix and were calculated with the regression equations in Winter (1990), based on the average stroke survivor height and weights reported in Olney and Richards (1996).

**Fig. 2.**
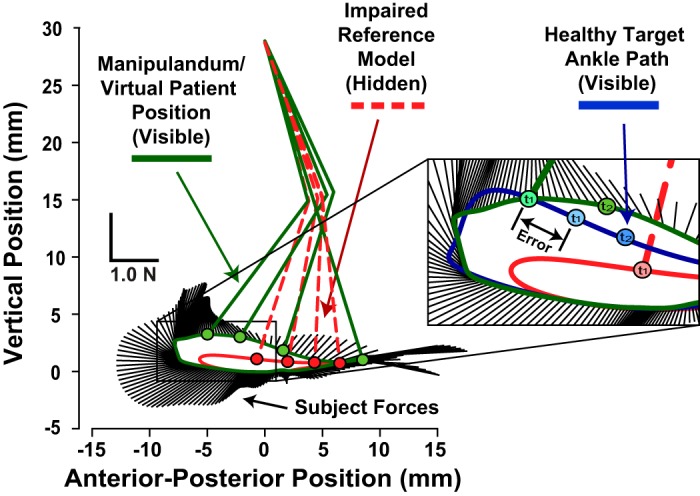
The virtual patient was rendered as an end-point impedance based on previously published kinematic and kinetic data from real stroke patients. The impaired reference model trajectory (red dashed leg and circles) represents the path the virtual patient’s leg took if unencumbered by external forces from the subject. Subjects did not see the reference model leg on the visual display. The leg associated with the actual manipulandum position (green leg and circles), which subjects saw, is an example of a deviated virtual patient locomotor pattern in response to subject forces. The same points in the gait cycle are shown for the actual and reference trajectories. The black lines radiating outward from the actual trajectory show a subject’s applied forces across a single step. *Inset* overlays the healthy target trajectory (blue), which subjects were asked to follow (*t*_1_ and *t*_2_ are time points).

The goal was to create a simple model that made subjects feel like they were holding onto the VP’s affected leg during treadmill locomotion with the manipulandum. It should be stressed that the model was not intended to be veridical but was designed to capture the basic features of the interaction. In the model, if a subject precisely followed along with the nominal (stroke based) movements of the VP’s leg, then the subject felt no interaction forces. However, if a subject tried to alter the VP’s motion, he/she began to feel the locomotor dynamics, which required the subject to generate appropriate forces to maintain the VP’s altered state (Fig. 2). The more severely the subject deviated the kinematics, the greater the effect. To achieve this behavior a mass-spring-damper impedance model was created, such that the subject felt a resistance (or assistance) that depended on gravitational, velocity, and inertia-dependent joint torques and how far the VP deviated from the reference stroke model kinematics, given by

(2) $$T=I\left(\theta \right){\overset{¨}{\theta }}_{\text{err}}+V\left(\theta ,\overset{˙}{\theta }\right){\overset{˙}{\theta }}_{\text{err}}+G\left(\theta \right)\theta_{\text{err}}$$

In *Eq. 2* the quantities ${\ddot{\theta }}_{\text{err}}$, ${\overset{˙}{\theta }}_{\text{err}}$, and **θ**_err_ refer to the vectors representing the difference between the hip and knee angular kinematics of the stroke reference model and the actual state of the VP as controlled by the subject with the manipulandum. For implementation, the joint torques were transformed to end-point forces at the point of interaction (the ankle) with the inverse transpose of the Jacobian **J**^−T^ (see appendix for **J**^−T^), such that

(3) $$F_{\text{D}}=J^{-\text{T}}T$$

where **F**_D_ = [F_D,_*_x_*,F_D,_*_y_*]*^T^* are the anterior-poster and vertical components of the to-be-rendered VP end-point force, respectively. The subscript D denotes that these are forces due to rigid body dynamics (as opposed to muscular; see *Neuromuscular contributions*).

#### Neuromuscular contributions.

**Fig. 3.**
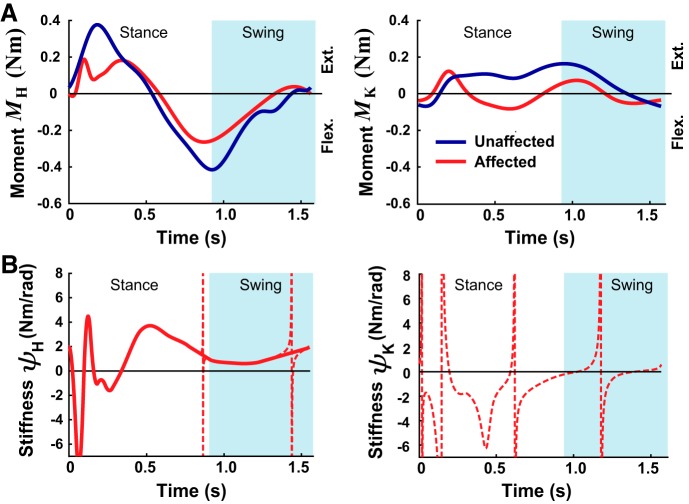
Data used to estimate muscular stiffness contributions. *A*: hip (*M*_H_) and knee (*M*_K_) joint moments from existing experimental data from the unaffected and affected sides of patients with stroke. *B*: muscular spring stiffness (ψ) estimated from hip and knee moment vs. angle. Discontinuities in the hip stiffness data (thin lines) were removed and linearly interpolated as shown. Since the knee spring stiffness makes relatively little contribution during swing (if discontinuity is removed), it was excluded from the virtual patient model.

If a subject tries to make the VP deviate from the nominal stroke trajectory, he/she should also feel forces reflecting the torques produced by the VP’s muscles. Although muscular torques are relatively small during leg swing, they are nonnegligible during push-off. Neuromuscular effects were represented by a muscular spring stiffness ***K***_SPR_, geometric stiffness ***K***_GEO_, and damping ***B***. Contributions from ***K***_SPR_ depended on the muscular spring stiffness coefficients ***ψ*** *=* [ψ_h_,ψ_k_]*^T^*, where ψ_h_ is the hip spring stiffness and ψ_k_ is the knee spring stiffness (see appendix for ***K***_SPR_ details). Values for ***ψ*** were obtained by computing the derivative of the internal hip and knee joint moments (Fig. 3*A*) with respect to the joint angles, based on the stroke patient data from Olney and Richards (1996). Discontinuous portions were removed and linearly interpolated (Fig. 3*B*). Because of the irregular nature of ψ_k_ and the fact that ψ_k_ was close to zero during most of the swing (ignoring the discontinuity), only the hip muscular stiffness was included in ***K***_SPR_. Although physiologically there is also a coupling stiffness from multiarticular muscles, this was excluded because of the lack of accurate experimental data. While ***K***_SPR_ reflected the springlike action of muscles, ***K***_GEO_ modeled how the limb end-point stiffness is affected by skeletal geometry in the presence of a contact force (English 1999). In general, ***K***_GEO_ is largest when the leg is relatively straight and increases in proportion to the contact force; the latter is reflected by the muscle-generated hip and knee joint torques: M_h_ and M_k_, respectively (these were obtained from the Olney and Richards data). An end-point contact force orientated toward the hip is less stable, i.e., acts like a hardening spring, compared with one that is oriented away from the hip, i.e., acts like a softening spring (equations describing ***K***_GEO_ can be found in appendix). Finally, muscular damping ***B*** was added to account for the viscosity-like effects of skeletal muscle mechanical properties. The damping was inversely proportional to the ankle velocity and ***K***_SPR_ (see appendix). These stiffness and damping effects can be expressed in equation form as an additional set of end-point forces:

(4) FM=(K + SPRKGEO)perr+Bp˙err

In *Eq. 4*, the quantity **F**_M_ = [F_M,_*_x_*,F_M,_*_y_*]*^T^* is a vector of the VP end-point force components due to muscular actions and ***p***_err_ and ${\overset{˙}{p}}_{\text{err}}$ are the difference vectors for the ankle position and velocity (stroke reference model minus actual VP state).

#### Treadmill model.

A simple treadmill model was incorporated into the simulation. A treadmill normal force **F**_N_ was created with a virtual sphere (radius = 7 mm in manipulandum workspace) that followed the VP ankle position and another of identical size that slid along a semicircular arc defining a modified virtual treadmill surface. The arc had a radius equal to 99.85% of the total VP leg length. The two spheres had a stiffness *k*_N_ = 2.0 N/mm, and if the spheres collided, i.e., if the VP’s ankle tried to penetrate the treadmill arc, the resistive force increased (see appendix for equation details). A second treadmill force **F**_S_ prevented slipping due to frictional forces and was modeled as a simple horizontal spring with stiffness *k*_S_ = 0.7 N/mm. The latter caused the manipulandum to pull the subject’s hand posteriorly (with respect to the VP), just as it would for a real patient on a treadmill. The net treadmill model force **F**_T_ was given by

(5) $$F_{T}=F_{N}+F_{S}$$

(6) FT=(kNp^err)+(kSperr)

where ***p̂***_err_ is the position of the VP’s ankle projected onto the surface of the virtual sphere defining the treadmill surface.

#### Net end-point force computation and scaling.

The force rendered by the manipulandum **F**_NET_ was the sum of the impedance forces computed for the rigid body dynamics, neuromuscular contributions, and the treadmill model, given by

(7) $$F_{\mathrm{NET}}=a\left(F_{D}+F_{M}+F_{T}\right)$$

where *a* is a scaling factor determined through experimentation (*a* = 1/12). This value was chosen so that the forces required to move the VP into a “healthy” trajectory were small enough (1–2 N) to prevent user fatigue and/or exceeding the manipulandum capabilities. Note that these forces are at least an order of magnitude less than the interaction forces previously reported by Galvez and colleagues (Galvez et al. 2005) for a therapist manipulating the gait of a real human patient with a spinal cord injury (American Spinal Injury Association impairment grade D).

#### Online processing of VP kinematics.

The impedance model requires online computation of the joint angular velocities and accelerations. Since the stroke reference kinematics are derived analytically, they are noise free. However, the manipulandum kinematics are encoder based and were therefore smoothed online to prevent noise amplification during numerical differentiation. First, ***θ*** was smoothed with a moving-average filter (MAF) using the prior 350 samples, which equates to a first-order low-pass filter with a ~2 Hz cutoff at the simulation sampling rate of 1,500 Hz. Next, a first-order adaptive windowing (FOAW) algorithm (Janabi-Sharifi et al. 2000) was used to calculate angular velocity ($\overset{˙}{\theta }$) with a 10-sample window (noise threshold = 0.05 rad) followed by smoothing with a 16-sample MAF. Acceleration ($\overset{¨}{\theta }$) was similarly estimated with FOAW and then smoothed with a 10-sample MAF. Although necessary, the online smoothing had the disadvantage that it introduced a delay in the VP position. Because of this, when the joint impedances were calculated the VP state was differenced against a delayed version of the reference state (delay = 160 ms).

### Experimental Approach

#### Subjects.

Seventeen young adults [*n* = 17; 13 women, 4 men; 16 right handed, 1 left handed, 27.1 yr (SD  4.4); 67.2 kg (SD 12.0) 1.7 m (SD 0.07)] participated in the study. All subjects were healthy and free from neurological and musculoskeletal impairments that affect the control of their upper extremity and gave their written informed consent. The study was approved by the Northeastern University Institutional Review Board.

#### Visual and auditory feedback and task instructions.

**Fig. 4.**
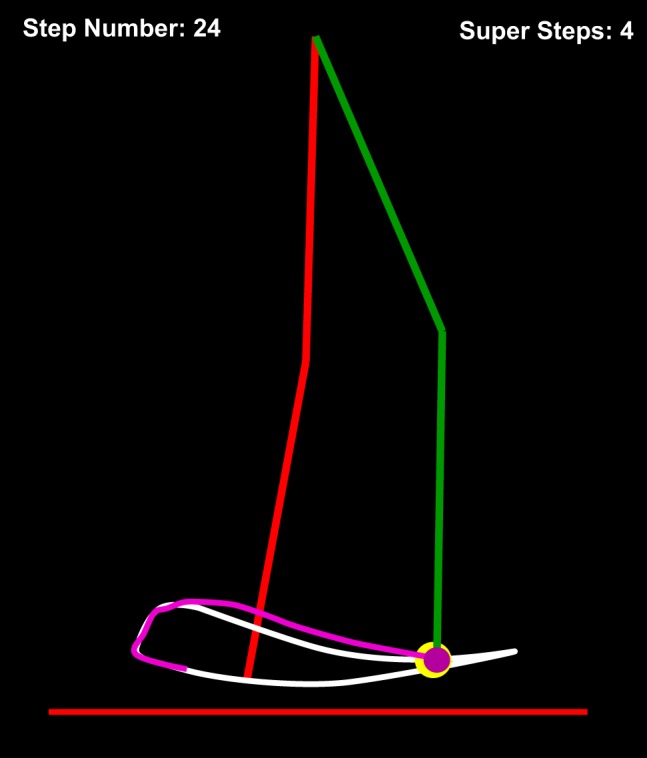
Example of visual display. The magenta dot represents the virtual patient’s (VP’s) right ankle joint (affected leg; green). The right ankle represents the point of physical interaction for subjects. The red unaffected leg serves as a visual reference. The yellow dot moves along the white target healthy trajectory; subjects were asked to make the VP’s ankle follow this dot. In the image, the target dot is yellow because the subject is very close to it; otherwise it turns white. A magenta tail followed the ankle to give additional performance feedback.

A monitor displayed a stick-figure representation of the VP’s lower body, and subjects saw both the unaffected and affected legs (Fig. 4). Subjects could not control the unaffected side, which always moved in a healthy gait pattern based on the previously recorded patient locomotor data. The manipulandum moved as if it was attached to the ankle of the VP’s affected leg. The thigh and shank segment positions were calculated online with the fixed hip position, ankle position, and inverse kinematics. The target ankle trajectory, which was either the healthy trajectory or a generalization target trajectory (see below for more details), was shown as a white line. During task practice, a white target sphere moved along the target ankle trajectory, showing where the VP’s ankle should be throughout the gait cycle. Subjects were instructed to practice making the VP’s ankle follow this sphere. The white sphere turned yellow whenever the instantaneous tracking error was <1.25 mm. To provide additional kinematic error information, a thin magenta “tail” followed the VP’s instantaneous ankle position, showing its displacement over the previous 1 s. This was particularly helpful in allowing subjects to gauge their performance during the relatively fast swing phase of gait. For additional pacing information, a “ding” was played each time the target sphere reached the end of the gait cycle at heel strike. A chime sound was played for each step that had a root-mean-squared error (between actual and target ankle positions) < 1 mm (called “super steps”), which were tallied on the screen.

#### Experimental setup.

Subjects sat in a chair behind a desk with the manipulandum and visual display in front of them on the desk. Subjects rested the middle of their dominant forearm on the edge of the desk and grasped the manipulandum with a writing implement grip.

#### Dynamics conditions.

In an impaired-dynamics condition, subjects felt forces that pulled their hand toward the nominal stroke patient-based locomotor trajectory as if holding onto the ankle of a (miniature) patient locomoting on a treadmill. In a null-dynamics condition, the locomotor dynamics were turned off, so subjects felt no forces when the VP’s leg was off the treadmill. The null condition controlled for tracking-related practice effects, i.e., so that when subjects experienced the impaired dynamics they were accustomed to the visual feedback and proficient at maintaining the appropriate locomotor timing. In both conditions the treadmill model remained in effect, i.e., the leg could not push through the treadmill and there was a horizontal force that dragged the leg rearward.

#### Healthy target trajectory practice.

**Fig. 5.**
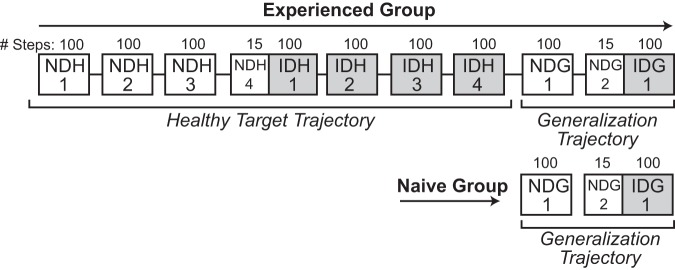
Schematic of experimental protocol for experienced and naive subject groups. NDH, null-dynamics/healthy target trajectory; IDH, impaired-dynamics/healthy target trajectory; NDG, null-dynamics/generalization trajectory; IDG, impaired-dynamics/generalization trajectory. Each 100-step NDH/NDG trial had 8 force-channel steps randomly inserted, and each IDH/IDG trial had 4 force-channel steps and 4 null-dynamics steps randomly inserted.

Subjects practiced making the VP’s affected leg follow the healthy target ankle trajectory (i.e., the 180° phase-shifted kinematics of the unaffected leg) for the majority of the experimental session (Fig. 5). They first performed the healthy target task for 100 steps in the null-dynamics condition (NDH1), and this was repeated twice more (NDH2 and NDH3). In the next trial, subjects practiced with null dynamics for 15 steps (NDH4), and on *step 16* the impaired locomotor dynamics were turned on and remained on for another 100 steps (IDH1). In the next three trials (IDH2–IDH4) subjects continued to practice making the VP move in the healthy locomotor pattern while compensating for the impaired locomotor dynamics (100 steps/trial).

#### Generalization test (silly walk).

An internal model of locomotor dynamics should allow participants to generate appropriate forces to move the VP into a variety of kinematic states, including those that differ from the ones practiced (Conditt et al. 1997). To test generalization, the kinematic goal of the task was changed while the underlying dynamics (the governing equations of motion) were kept the same. This was achieved by changing the target kinematic trajectory from the healthy target to the generalization target (silly walk). The latter deviated from the characteristic pendular motion of leg swing and required an exaggerated step height and a different kinematic profile (see Fig. 1). The generalization target practice lasted for 100 steps under null dynamics (NDG1), followed by one block with 15 null field steps (NDG2) and then 100 impaired-dynamics steps (IDG1; see Fig. 5).

#### Catch steps.

Randomly applied force channels (Scheidt et al. 2000) and null-dynamics (Shadmehr and Mussa-Ivaldi 1994) catch steps were used to assess internal model adaptation during practice. For the force-channel steps, a stiff spring applied forces to create a channel that made the manipulandum (VP’s ankle) follow the target trajectories (healthy or generalization). The forces were orthogonal to the target trajectories. During null-dynamics steps the impaired dynamics were turned off, i.e., the treadmill remained, but during the swing phase subjects moved freely in the air. The catch steps were initiated while the VP’s foot was on the treadmill (affected leg), remained in effect throughout the swing phase, and turned off after the VP’s foot returned to the treadmill. Since kinematic errors were minimal during treadmill contact, subjects could not feel the catch steps initiating. Each impaired-dynamics trial had four force channels and four null-dynamics steps randomly inserted, and each 100-step null-dynamics trial had eight force channels randomly inserted (additional force-channel steps were inserted in lieu of null-dynamics steps).

The experiment used both types of catch steps because they provide a window into a subject’s forward motor plan yet differ in the specifics of the assay. The null-dynamics steps are useful because they allow substantial kinematic errors to develop, which reveals the consequences of a suddenly incorrect internal model (if one exists), which in turn engages a relatively rapid error correction system likely driven by muscle spindle feedback (Scheidt et al. 2000). On the other hand, force channels do not elicit a large error correction response, with much slower adaptation possibly driven by Golgi tendon organs and mechanoreceptors (Scheidt et al. 2000). This allows force channels to probe internal model output later in the swing phase of the locomotor cycle, which is of interest in the present study.

### Data Analysis

All analysis procedures were performed in MATLAB (version 9.4, R2018a; MathWorks, Natick, MA). Primary dependent variables included the VP tracking error, ankle kinematics, and the force applied by the subjects on the VP. The tracking error was calculated as the root-mean-squared Euclidean distance error between the manipulandum end-point position (VP’s ankle) and the locomotor target tracker. The tracker was the white/yellow sphere that followed the target trajectory showing where the VP’s ankle should be at each point in the gait cycle. For each trial the continuous data record was separated into individual strides with the maximum forward displacement of the ankle joint as the separation point (end of the swing phase). Each stride was normalized to a percentage of the stride cycle. Variables were averaged over either the last 30 steps for the 100-step conditions (NDH1–NDH3, IDH1–IDH4, NDG1, and IDG1) or over the last 10 steps for the 15-step conditions (NDH4 and NDG2). To capture early learning transients, the first five trials were analyzed separately for the beginning of the null-dynamics practice trials (NDH1 Early and NDG1 Early) and the beginning of the impaired-dynamics practice trials (IDH1 Early and IDG1 Early). Force-channel and null-dynamics responses were averaged within each trial for each subject. As subjects were imperfect at following the healthy trajectory even in the null-dynamics trials (see results), the average force-channel forces during the last null-dynamics healthy target trial (NDH3) were subtracted from those measured in each of the impaired-dynamics trials (IDH1–IDH4) for each subject. These are subsequently referred to as normalized force-channel steps. One subject was classified as an outlier based on very high performance errors compared with the other subjects [>*q3* + 2.7σ × (*q3* – *q1*), where σ is the standard deviation and *q1* and *q3* are the 25th and 75th percentiles, respectively, based on MATLAB function *boxplot.m*]; thus the analysis was completed with 16 subjects.

### Statistics

Separate repeated-measures analysis of variance (RM ANOVA) procedures were performed to determine whether there were significant practice effects during the initial null-dynamics healthy target practice trials (NDH1–NDH4) and impaired-dynamics practice trials (IDH1–IDH4) for tracking error, peak anterior-posterior and vertical ankle displacements, and peak subject-applied forces. Paired *t*-tests were used for other planned comparisons to determine whether the initial exposure to the impaired dynamics caused a significant change in the dependent variables in both the healthy target (NDH4 vs. IDH1 Early) and generalization target (NDG2 vs. IDG1 Early) conditions. Significance was at *P* < 0.05 for these statistical tests and all others. Statistics were performed in MATLAB and SPSS (version. 25; IBM, Armonk, NY).

Potential changes in the magnitude of the normalized channel forces with practice were examined at five time points across the swing phase of the gait cycle (60%, 70%, 80%, 85%, and 95%) with RM ANOVAs (across trials IDH1–IDH4). The finer-grained spacing between 80% and 95% was used because the ankle moves faster during this time. Practice effects for the angle of the channel force vectors were analyzed with circular statistics. The Cramer-Von Mises goodness of fit hypothesis test (Zar 1999) indicated that the angle data were not normally distributed; thus differences among the practice trials (IDH1–IDH4) were tested nonparametrically with the Moore test (Moore 1980) for paired circular data (Zar 1999). For the null-dynamics catch steps, a RM ANOVA tested for differences in the peak anterior-posterior and vertical trajectory errors with practice (across IDH1–IDH4).

### Generalization vs. Naive Participants

To assess generalization, a second new group of 10 subjects, called the naive group, was recruited [7 women, 3 men; all right handed, 26.5 yr (SD 5.4); 70.5 kg (SD 16.9); 1.7 m (SD 0.08)]. All subjects provided written informed consent as described above. This group began their practice session making the VP’s affected leg follow the generalization (silly walk) trajectory for 100 steps in the null field (NDG1). The naive group never practiced making the VP follow the healthy target trajectory (Fig. 5). NDG1 practice was followed by one block with 15 null field steps (NDG2) and 100 impaired-dynamics steps with the generalization target (IDG1). Force channels and null-dynamics steps were included as described for the first experiment. To differentiate between the groups, the original group of subjects who practiced the healthy walk task first are called the experienced group.

The generalization analysis compared the experienced and naive groups during the generalization trajectory practice to test whether the experienced group had an advantage and tested for differences in aftereffects. The dependent variables included those previously described: tracking error, force-channel forces, and null-trial deviations. Unpaired Welch’s *t*-tests (Welch 1947) were used for all between-group tests because of different group sizes, except for the circular data (force vector angles), which used Watson’s test (Watson 1962). For the force channels, differences in subjects’ applied force were assessed at five points in the gait cycle (55%, 57.5%, 60%, 65%, and 70%). This set of points differs from the healthy target points (60%, 70%, 80%, 85%, and 95%) because of timing differences, i.e., if the same healthy target time points were used for the generalization target they would be clustered at the very end of the swing. To control for multiple comparisons, the Benjamini-Hochberg step-up procedure (Benjamini and Hochberg 1995) was used to calculate adjusted *P* values (*P̃*) with a tolerable false discovery rate of 0.1, based on the recommendation of McDonald (2014) (p. 254–260). For the null steps, the average absolute tracking error during the null steps was computed for each subject and compared between the groups.

## RESULTS

### Task Performance

**Fig. 6.**
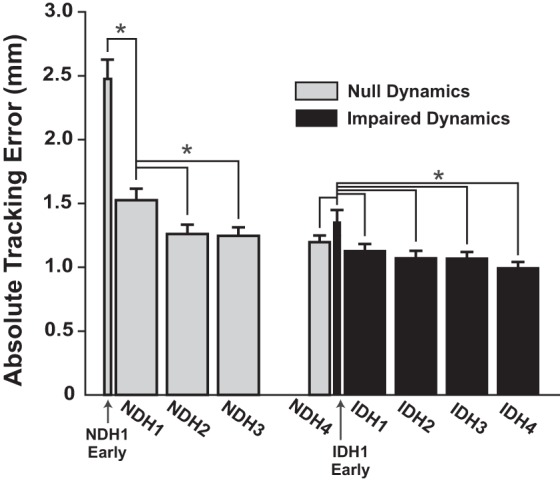
Tracking error for all subjects as they practiced making the virtual patient’s gait symmetric (healthy target). NDH, null-dynamics/healthy target; IDH, impaired-dynamics/healthy target. Means + SE shown. *Significance at *P* < 0.05.

Performance was quantified as the average tracking error throughout the gait cycle; the results are summarized in Fig. 6. For the initial null-dynamics/healthy target practice (NDH1–NDH4), subjects improved their tracking, as the RM ANOVA revealed a main effect of time (*F*[4, 60] = 39.6, *P* < 0.001). Error decreased significantly from the beginning to the end of the first practice block and decreased again at the end of the second block (from NDH1 Early to NDH2; *P* < 0.05 for each pairwise comparison). By the end of the initial null-dynamics/healthy target practice block, subjects were able to closely track the healthy trajectory, with mean errors of ~1.25 mm. When the stroke was first turned on (IDH1 Early) there was a small but significant increase in error (NDH4 vs. IDH1 Early: *P* = 0.038). Error quickly decreased by the end of the first stroke-dynamics/healthy target practice block (RM ANOVA for IDH trials: *F*[4, 60] = 7.7, *P* < 0.001; pairwise comparison for IDH1 Early vs. IDH2: *P* < 0.05) but did not exhibit further decreases after that. By the end of IDH4 (last 30 trials) the percentage of super steps was 53.5%  (SD 25.5). Silly walk results are reported in *Generalization vs. Naive Participants* below.

### Kinematics and Kinetics

**Fig. 7.**
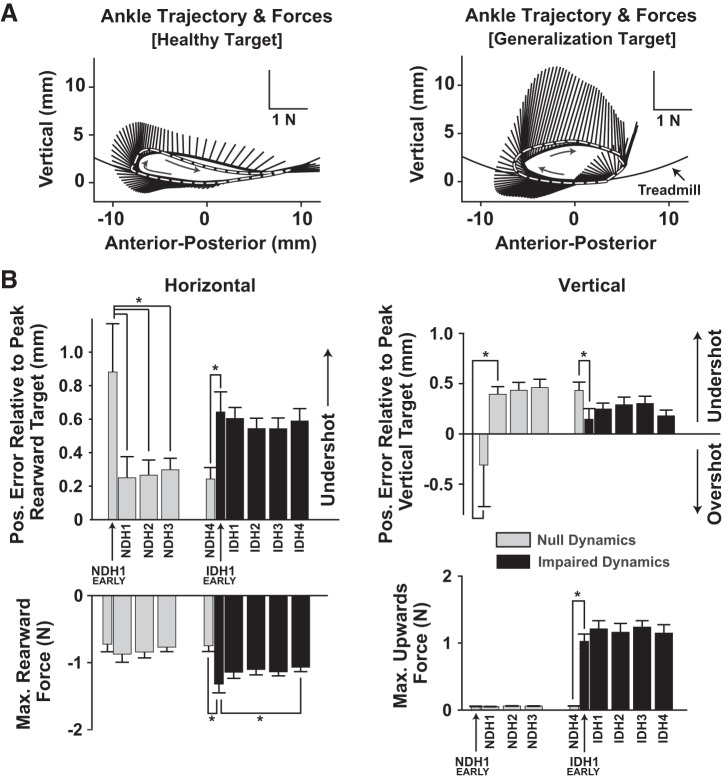
Changes in kinematic error and peak force magnitudes over practice. *A*: average ankle trajectory for the last 30 steps of the impaired-dynamics trials with healthy and generalization targets (IDH3 and IDG1, respectively). The target trajectory is overlaid as a dashed black-and-white line; subjects’ average applied forces are shown as lines radiating outward. *B*: maximum rearward and upward position errors (*top*) and subject-applied forces (*bottom*) during healthy trajectory practice (means + SE). NDH, null-dynamics/healthy target trajectory; IDH, impaired-dynamics/healthy target trajectory. *Significance at *P* < 0.05.

The path of the VP’s ankle during practice with the impaired dynamics is shown in Fig. 7*A*. To quantify adaptation, the maximum rearward and upward VP ankle displacements are shown across practice in Fig. 7*B*. These data show that subjects did not pull the leg far enough rearward (undershoot). This undershoot decreased rapidly during the NDH trials (RM ANOVA: *F*[4, 60] = 4.774, *P* = 0.002; pairwise comparisons show that NDH1 Early had more rearward undershoot than NDH1–NDH4: *P* < 0.05 for all). The undershoot became greater when transitioning to the IDH trials (NDH4 vs. IDH1 Early; *P* < 0.001) but did not change with further practice across IDH trials (RM ANOVA: *F*[4, 60] = 0.405, *P* = 0.804). In early NDH practice, subjects moved the VP’s ankle too high (overshoot), and this switched to an undershoot for the rest of NDH practice (RM ANOVA: *F*[4, 60] = 2.953, *P* = 0.027). When the stroke dynamics were turned on, vertical undershoot decreased (NDH4 vs. IDH1 Early; *P* = 0.022) but did not change with further practice across IDH (RM ANOVA: *F*[4, 60] = 1.766, *P* = 0.147).

Inspection of the average subject-applied force patterns show that, as expected, subjects pulled rearward and upward to match the locomotor targets during heel lift and initial leg swing (Fig. 7*A*). The peak posterior and upward forces were analyzed to assess adaptation (Fig. 7*B*). There were no differences in the peak posterior force across the NDH trials (RM ANOVA: *F*[4, 60] = 0.542, *P* = 0.705). When the stroke dynamics were turned on, the maximum posterior force increased (NDH4 vs. IDH1 Early; *P* = 0.014) and there was a small decrease across the IDH trials (RM ANOVA: *F*[4, 60] = 3.201, *P* = 0.019; pairwise comparison for IDH1 Early had greater posterior force than NDH4: *P* = 0.043). There were no practice-related changes in the peak vertical force across NDH (RM ANOVA: *F*[4, 60] = 1.908, *P* = 0.121). The peak vertical force increased when the stroke dynamics were turned on (NDH4 vs. IDH Early; *P* < 0.001) but did not change with additional IDH practice (RM ANOVA: *F*[4, 60] = 0.867, *P* = 0.489).

### Catch Steps

**Fig. 8.**
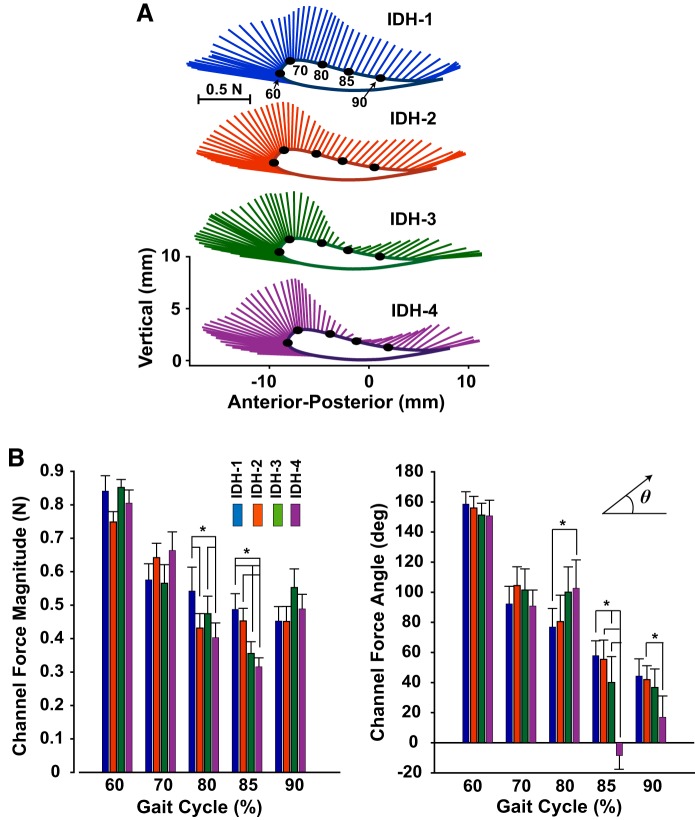
Patterns of force exerted by subjects against the force channels show refinements that take advantage of pendular locomotor dynamics. *A*: forces subjects applied to the force channels averaged across subjects over the gait cycle for each of the 4 impaired-dynamics/healthy target practice trials (IDH1–IDH4). The average path of the virtual patient’s ankle is shown. Each plot represents the average over the 4 force channel steps within an IDH trial, with the forces subjects exerted against the channel during the last null-dynamics healthy target trial (NDH3) subtracted. *B*: subject-applied force magnitude and direction (angle) at different points in the gait cycle (shown as dots along the ankle trajectories in *A*). Each bar shows the mean + SE across subjects. *Significance at *P* < 0.05.

The normalized force-channel forces, i.e., with null-dynamics effects subtracted out, were nonzero (Fig. 8*A*), indicating that subjects pushed against the channel walls. How subjects pushed against the channel changed with practice. This effect was quantified at five time points across the swing phase of gait. During midswing there was a progressive decrease in the magnitude of subject-applied forces, at both 80% (RM ANOVA: *F*[3, 45] = 4.5, *P* = 0.009) and 85% (RM ANOVA: *F*[3, 45] = 13.9, *P* < 0.001) of the gait cycle (Fig. 8*B*). There were also changes in the angle of force application with practice (Fig. 8*B*), with forces becoming more vertical at 80% (IDH1 vs. IDH4: *R*′ = 1.224, *P* < 0.025) and more anterior at 85% and 90% (at 85% IDH1 vs. IDH4: *R*′ = 1.515, *P* < 0.001; at 90% IDH2 vs. IDH4: *R*′ = 1.043, *P* < 0.05).

**Fig. 9.**
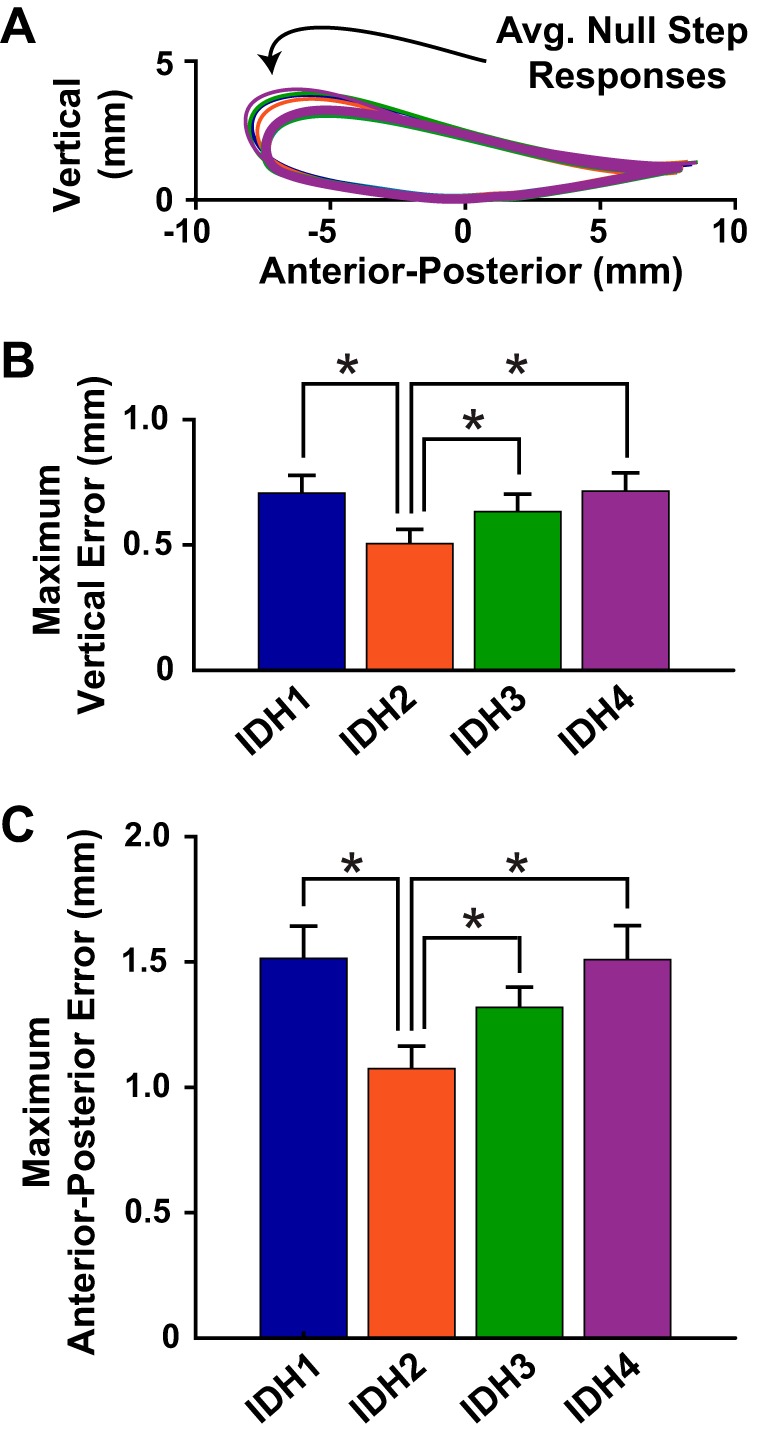
Errors made when the stroke locomotor dynamics were suddenly turned off showed adaptation with practice. *A*: virtual patient ankle trajectories during impaired- (thick lines) and null-dynamics (thin lines) steps. *B*: maximum vertical errors during null-dynamics steps. *C*: maximum anterior-posterior errors. IDH, impaired dynamics/healthy target trajectory. Means + SE. *Significance at *P* < 0.05.

When the impaired dynamics were turned off during the random null-dynamics steps, subjects made kinematic errors (Fig. 9*A*). During each IDH practice trial the peak null-step vertical (Fig. 9*B*) and anterior-posterior (Fig. 9*C*) errors were significantly larger than zero (*P* < 0.01). The RM ANOVA revealed a main effect of time; the peak errors changed with practice (anterior-posterior error: *F*[3, 45] = 5.578, *P* = 0.002; vertical error: *F*[3, 45] = 4.046, *P* = 0.012). Pairwise comparisons showed a significant drop in the anterior-posterior and vertical errors from IDH1 to IDH2 and rising again to IDH3 and IDH4 (*P* < 0.05 for all; note that for IDH2 vs. IDH3 vertical error the test is borderline, with *P* = 0.048). 

### Generalization vs. Naive Participants

**Fig. 10.**
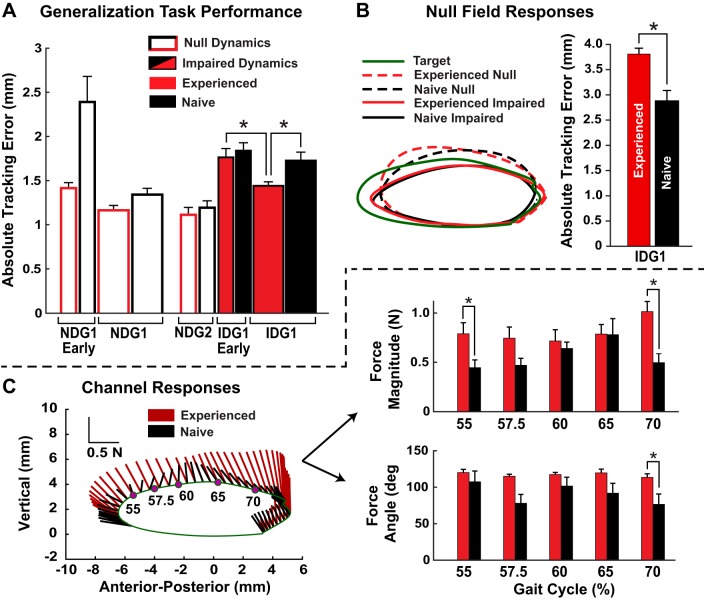
Prior exposure to stroke locomotor dynamics was associated with improved task performance and different aftereffects. The experienced group first practiced making the virtual patient (VP) follow a healthy locomotor trajectory and then practiced making the VP follow a generalization trajectory. A second naive group only practiced the generalization trajectory. *A*: tracking error results. *B*: average responses to the 4 null trials, in which the impaired dynamics were unexpectedly turned off for 1 step. *C*: averaged force applied by the subjects during the force channel trials. NDG, null dynamics with generalization target; IDG, impaired dynamics with generalization target. Means + SE shown. *Significance at *P* < 0.05.

In contrast to the experienced group, the naive group started practice tracking the generalization trajectory (Fig. 5). After practicing in the null-dynamics field, the naive group had a tracking proficiency similar to the experienced group (Fig. 10*A*; NDG2 experienced vs. naive: *P* = 0.570). The initial decrement in performance in response to the impaired dynamics being turned on was similar for both groups (no between-group difference in change from NDG2 to IDG1 Early; *P* = 0.574). However, with continued practice the experienced group decreased their tracking error more than the naive group (change from IDG1 Early to IDG1; *P* = 0.030) and had a smaller tracking error at the end of generalization trajectory practice (between-group IDG1 comparison; *P* = 0.004). This suggests generalization in the experienced group.

Analysis of the force-channel data in the silly walk condition showed differences in the patterns of forces between the experienced and naive groups (Fig. 10*B*). The normalized force magnitude (with null-dynamics channel forces subtracted) was greater in the experienced group early after the VP’s foot left the ground at 55% of the gait cycle (*P* = 0.032; *P̃* = 0.080) but not at 57.5% (*P* = 0.077; *P̃* = 0.128). Note that in this case the adjusted *P* value (*P̃*) is derived from the Benjamini-Hochberg step-up procedure and is compared against the specified tolerable false discovery rate (i.e., if *P̃* < 0.1, then the test is significant). The experienced group channel forces were larger and more vertical right before the foot was placed back on the ground (at 70%: force magnitude *P* = 0.001 and *P̃* = 0.005; force direction *P* = 0.012; *P̃* = 0.060). During the random null-dynamics steps both experienced and naive groups made errors, pulling the leg too high above the generalization target trajectory (Fig. 10*C*). However, the experienced group made larger errors during these steps compared with the naive group (*P* = 0.002). Together, these results indicate larger aftereffects in the experienced group.

## DISCUSSION

### Main Findings

This study sought an answer to the question of how humans learn to modify the locomotor dynamics of a virtual patient (VP). This question is nontrivial because of the complexity of the dynamical interaction, i.e., it is continuous and involves position-, velocity-, and acceleration-dependent forces. A novel interactive locomotor simulator was developed that reproduced the dynamics of hemiparetic gait. In the simulator, subjects used a small manipulandum to physically interact with the VP’s affected leg and attempted to make its locomotor kinematics more symmetric. The hypothesis was that subjects would accomplish this task by learning an internal model of the VP’s locomotor dynamics. The alternative hypothesis was that in such dynamically complex situations humans default to other strategies that do not rely on explicit dynamics models. The results provide evidence that supports the internal model hypothesis: the presence of aftereffects (persistence of feedforward motor plans despite altered dynamics) and generalization of manipulation skill. Moreover, the results demonstrate how internal locomotor models develop over time, shifting from a general to a specific strategy that required less assistive force by taking advantage of the pendular dynamics of the VP’s leg. In other words, subjects seemed to transition from a more heavy-handed position-control approach to one that more closely resembled the assist-as-needed approach commonly used in robotic gait rehabilitation (Cai et al. 2006).

### Task Performance and Adaptation

To achieve a low tracking error, subjects had to make the VP’s affected leg follow a healthy locomotor pattern. The latter was based on the VP’s unaffected leg kinematics, and therefore successful task performance made the VP’s locomotor kinematics symmetric. This required subjects to appropriately compensate for impedance forces that tried to maintain the VP’s nominal impaired locomotor pattern. With practice, subjects became proficient and were able to reduce their tracking error to ~1.25 mm on average with peak forces of ~1 N. Although the average tracking error was small, subjects did not precisely follow the healthy target trajectory and instead rounded off/approximated certain portions. This behavior could be because the curvature of the target ankle trajectory exceeded the ability of subjects to follow with the requisite velocity because of the one-third power law (Lacquaniti et al. 1983). Alternatively, it could be due to high centripetal accelerations encountered during the heel lift and swing initiation, which are higher than would be encountered in real life because of the smaller radius of curvature in the VP simulation. In the generalization task, errors and forces increased by ~50% and subjects did not pull enough posteriorly and upward to match the target trajectory. This was evident in both the experienced and naive groups and could be because subjects had only a single 100-step practice trial with the impaired dynamics turned on while tracking the generalization target.

### Internal Representations During Task Adaptation

The central hypothesis was that humans develop internal models of locomotor adaptation. An alternative is a brute-force strategy, i.e., subjects could increase their arm’s end-point impedance by using antagonistic coactivation to overpower any perturbations from the VP (Hogan 1984). Normally, a downside of this strategy is that it incurs a large energetic cost (Falconer and Winter 1985; Macaluso et al. 2002), but in this study the magnitude of the forces exerted by subjects was relatively small (~1 N). Nevertheless, the data rule out a pure brute-force strategy because subjects made significant errors during the random null-dynamics catch steps, which would not be expected if subjects exhibited a high nondirectional arm stiffness. Notably, the errors on null-dynamics steps changed with practice. Early on, the size of the null-trial errors decreased before increasing again. This suggests that early in practice subjects could have employed a partial brute-force “stiffness” strategy when the internal model was still developing (Milner and Franklin 2005). In a rehabilitation context, this resembles the early guidance approach used in robotic rehabilitation, which made use of strong nonbackdrivable motors to force a patient’s leg to follow a desired trajectory (Hornby et al. 2005). With practice, subjects’ internal models became stronger, decreasing the need to rely on muscle viscoelasticity to reduce task errors (Osu et al. 2002). The latter more closely resembles the assist-as-needed robotic rehabilitation approach (Cai et al. 2006).

A second argument against the use of a pure brute-force strategy is that subjects applied significant forces against the force channels and these responses were modified with practice. Since the force channels were imposed unexpectedly, they (approximately) reflect the forward motor plan and provide insight into how subjects’ internal models adapted. Of particular significance is that the forces applied to the channel changed with practice to take better advantage of the VP’s locomotor physics. Specifically, subjects learned to apply less force during midswing when the pendular dynamics of the leg would naturally tend to progress the leg forward. This is consistent with prior studies showing that humans learn to take advantage of the intrinsic dynamics of an external system, for example, by forcing a system close to its resonant frequency (Hatsopoulos and Warren 1996; Holt et al. 1991; Huang et al. 2007; White et al. 2008) or with exposure to artificial negative viscosity (Huang et al. 2010), and the idea that one of the optimization criteria for human sensorimotor control is minimizing energy expenditure or effort (Alexander 1997; Todorov and Jordan 2002). However, this study also shows that exploiting natural dynamics *1*) may occur at the level of internal models and *2*) is observed for a task with complex rhythmic dynamics (locomotion).

Another alternative hypothesis is that subjects used a rote memorization strategy, in which they learned to predict forces explicitly as a function of time (Conditt et al. 1997; Conditt and Mussa-Ivaldi 1999). While the null-dynamics and force-channel probes cannot rule this out, the generalization test can: an internal model should facilitate generalization, but rote memorization should not (Conditt et al. 1997). This was tested by asking subjects to make the VP walk in a different generalization trajectory—the silly walk. If learning generalized, subjects should perform as well or better at the generalization trajectory compared with naive subjects who did not previously practice the healthy trajectory. The results showed that the naive group improved less than the experienced group, which suggests that the experienced subjects were not just working from a memorized “tape recording” of motor commands but were able to use their implicit knowledge of the VP locomotor dynamics to adapt to the new task requirement (i.e., they generalized learning). This is consistent with other reports showing generalization when subjects adapt to a new movement pattern that covers similar states (Conditt et al. 1997; Shadmehr and Mussa-Ivaldi 1994) and when training with an artificial robot-induced negative viscosity (Huang et al. 2010). However, it is worth noting that other studies have shown more limited generalization when subjects are asked to extrapolate to more novel situations (Gandolfo et al. 1996; Goodbody and Wolpert 1998) and when learning force fields with explicit time dependencies (Conditt and Mussa-Ivaldi 1999). Thus in future work it would be interesting to test the extent of generalization for VPs modeled after different patient populations (e.g., Parkinson’s disease or spinal cord injury), which could have vastly different walking speeds in addition to differences in neuromuscular control.

### Extensions to Rehabilitation (Caveats)

The advantage of having subjects train the VP in a locomotor simulator is one of experimental control: the VP’s dynamics can be precisely specified and instantaneously manipulated. However, the degree to which the results extend to real patients is presently unknown. Although the VP model included many features of real locomotor dynamics, including inertial effects, geometric constraints, and muscular contributions, it was not intended to be a precise model of human locomotion. For example, the knee joint muscular spring stiffness was excluded, the knee-hip coupling stiffness was absent, and the VP conspicuously lacked a foot. The latter required some creativity concerning the leg-treadmill interface, which was made semicircular to make up for the absent degree of freedom (the ankle). Because the model only operated in the sagittal plane and the hip joint could not translate, the model could not capture the circumduction and hip-hiking strategies that some patients employ to prevent toe dragging (Davids 1992). It is also important to note that the simulator turned a gross-motor task, therapist-guided locomotor rehabilitation, into a fine-motor task. Compared with interactions with real patients, the level of manipulation force required from the subjects was reduced by an order of magnitude, and the spatial extent of movement shrank by 96.5%. Also, real rehabilitation is a cooperative human-human interactive task (Jarrassé et al. 2012; Sawers and Ting 2014) and real patients would be expected to adapt over the course of training. Therefore, the results of this study should be interpreted in the context of the hypothesis, i.e., understanding the sensorimotor processes used by humans when learning how to manipulate a virtual model of locomotor dynamics, and not extrapolated to real patients at the present time.

### A Future Platform for Understanding Locomotor Rehabilitation

The locomotor simulator presents an intriguing set of potential experimental manipulations that could increase our understanding of locomotor rehabilitation. However, this may require increases in the sophistication of the VP’s neural control. For example, in the present study, on purpose, the VP always tried to walk in the same stroke-based asymmetric locomotor pattern and never adapted its behavior during the course of the experiment. This clearly neglects the richness of human sensorimotor control and the stride-to-stride variability that reflects human control processes and the natural environment (Hausdorff 2007). Programmatically, it would be simple to add different noise structures to the model, e.g., one could add 1/f noise to create long-range correlations in the VP’s gait (Hausdorff et al. 1996). It could also be instructive to allow the VP to adjust its actions based on the errors it makes, which could, in turn, be influenced by the actions of a therapist. For example, the VP could be given an explicit control policy that could optimize maintaining stability (Cajigas et al. 2017) or other costs that might be relevant to patient populations, such as pain (Arendt-Nielsen and Graven-Nielsen 2008; Boudreau et al. 2010) or safety (Hasson and Sternad 2014). This may help answer new and relevant theoretical questions, such as how to guide patients with different and possibly shifting motor priorities. It could also serve as a practical tool that allows clinicians to experience therapeutic interactions with various virtual gait disorders before delivering care to real patients. Of course, drawing conclusions about patient control policies will require models that are validated against the populations in question.

### Conclusions

This study developed an interactive locomotor simulator that allowed subjects to manipulate the gait patterns of a virtual patient. The aim was to understand the sensorimotor processes used by humans when learning how to manipulate locomotor dynamics. The results showed that, despite the complexities of the interaction, subjects nevertheless developed and used internal models that generalized to a different locomotor pattern. With practice the internal model became more refined to take advantage of the pendular dynamics of locomotion. With further advancements to the neuromusculoskeletal model, the locomotor simulator may serve as a useful tool for increasing our understanding of locomotor rehabilitation and inform both human and robotic therapy for more effective training outcomes.

## GRANTS

This work was funded in part by a Northeastern University seed/proof of concept grant (C. J. Hasson).

## DISCLOSURES

No conflicts of interest, financial or otherwise, are declared by the authors.

## AUTHOR CONTRIBUTIONS

C.J.H. conceived and designed research; C.J.H. and S.E.G. performed experiments; C.J.H. and S.E.G. analyzed data; C.J.H. and S.E.G. interpreted results of experiments; C.J.H. prepared figures; C.J.H. and S.E.G. drafted manuscript; C.J.H. and S.E.G. edited and revised manuscript; C.J.H. and S.E.G. approved final version of manuscript.

## Figures and Tables

**Fig. A1.**
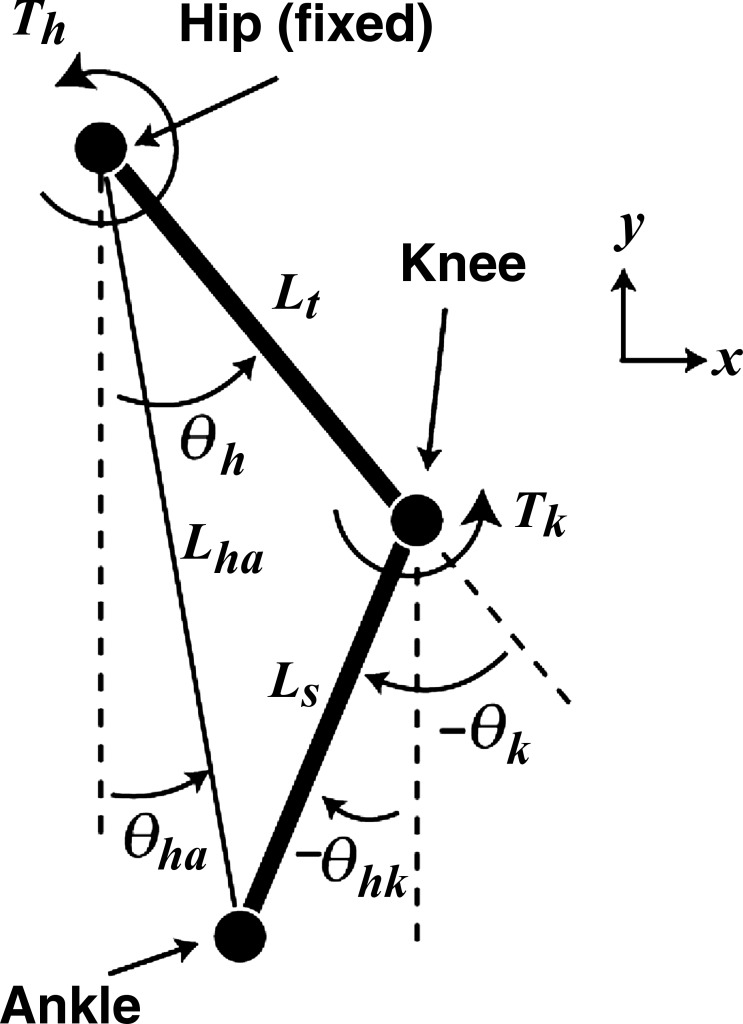
Schematic showing segment length (*L*), joint angle (θ), and joint torque (T) conventions. h, Hip; ha, hip to ankle; hk, hip to knee; k, knee; s, shank; t, thigh.

## APPENDIX: MATRICES ASSOCIATED WITH VIRTUAL PATIENT MODEL

For simplicity, all equations below were derived in the first quadrant and rotated 90° clockwise into a physiologically realistic orientation for locomotion (Fig. A1).

### Rigid Body Dynamics

$$I\left(\text{θ}\right)=\left[\begin{array}{cc} {\text{I}}_{\text{t}}+{\text{I}}_{\text{s}}+m_{\text{t}}r_{\text{t}}^{\text{2}}+m_{\text{s}}r_{\text{s}}^{\text{2}}+m_{\text{s}}L_{\text{t}}^{\text{2}}+2m_{\text{s}}L_{\text{t}}r_{\text{s}}\cos{\text{θ}}_{\text{k}} & {\text{I}}_{\text{s}}+m_{\text{s}}r_{\text{s}}^{\text{2}}+m_{\text{s}}L_{\text{t}}r_{\text{s}}\cos{\text{θ}}_{\text{k}}\\ {\text{I}}_{\text{s}}+m_{\text{s}}r_{\text{s}}^{\text{2}}+m_{\text{s}}L_{\text{t}}r_{\text{s}}\cos{\text{θ}}_{\text{k}} & {\text{I}}_{\text{s}}+m_{\text{s}}r_{\text{s}}^{\text{2}} \end{array}\right]$$

$$V\left(\text{θ},\overset{˙}{\theta }\right)=\left[\begin{array}{cc} -m_{\text{s}}L_{\text{t}}r_{\text{s}}\sin{\text{θ}}_{\text{k}}{\overset{˙}{\theta }}_{\text{k}} & -2m_{\text{s}}L_{\text{t}}r_{\text{s}}\sin{\text{θ}}_{\text{k}}{\overset{˙}{\theta }}_{\text{k}}\\ -m_{\text{s}}L_{\text{t}}r_{\text{s}}\sin{\text{θ}}_{\text{k}}{\overset{˙}{\theta }}_{\text{h}} & 0 \end{array}\right]$$

$$G\left(\text{θ}\right)=-g\left[\begin{array}{c} m_{\text{t}}r_{\text{t}}\sin{\text{θ}}_{\text{h}}+m_{\text{s}}l_{\text{t}}\sin{\text{θ}}_{\text{h}}+m_{\text{s}}r_{\text{s}}\cos{\text{θ}}_{\text{hk}}\\ m_{\text{s}}r_{\text{s}}\sin{\text{θ}}_{\text{hk}} \end{array}\right]$$

### Muscular Spring Stiffness Expressed at Ankle End Point (English 1999)

$$K_{\text{SPR}}=\left[\begin{array}{cc} k_{xx}^{\text{S}} & k_{xy}^{\text{S}}\\ k_{yx}^{\text{S}} & k_{yy}^{\text{S}} \end{array}\right]$$

$$k_{xx}^{\text{S}}={\left(\frac{1}{L_{\text{t}}L_{\text{s}}\sin{\text{θ}}_{\text{k}}}\right)}^{2}{\left(L_{\text{s}}\sin{\text{θ}}_{\text{hk}}\right)}^{2}\Psi_{\text{h}}$$

$$k_{xy}^{\text{S}}={\left(\frac{1}{L_{\text{t}}L_{\text{s}}\sin{\text{θ}}_{\text{k}}}\right)}^{2}L_{s}^{2}\sin{\text{θ}}_{\text{hk}}\cos{\text{θ}}_{\text{hk}}\Psi_{\text{h}}$$

$$k_{yx}^{\text{S}}=k_{xy}^{\text{S}}$$

$$k_{yy}^{\text{S}}={\left(\frac{1}{L_{\text{t}}L_{\text{s}}\sin{\text{θ}}_{\text{k}}}\right)}^{2}{\left(L_{\text{s}}\cos{\text{θ}}_{\text{hk}}\right)}^{2}\Psi_{\text{h}}$$

### Muscular Geometric Stiffness Expressed at Ankle End Point (English 1999)

$$K_{\text{GEO}}=\left[\begin{array}{cc} k_{xx}^{\text{G}} & k_{xy}^{\text{G}}\\ k_{yx}^{\text{G}} & k_{yy}^{\text{G}} \end{array}\right]$$

$$k_{xx}^{\text{G}}=\left(\frac{1}{L_{\text{t}}^{\text{2}}L_{\text{s}}^{\text{2}}\sin{\text{θ}}_{\text{k}}^{3}}\right)\left(L_{\text{s}}^{2}\sin{\text{θ}}_{\text{hk}}^{2}\cos{\text{θ}}_{\text{k}}+L_{\text{s}}L_{\text{t}}\sin{\text{θ}}_{\text{hk}}^{2}\right)M_{\text{h}}-\left(L_{\text{d}}^{2}\cos_{\text{d}}^{2}\cos{\text{θ}}_{\text{k}}+L_{\text{s}}L_{\text{t}}\sin{\text{θ}}_{\text{k}}^{2}\right)M_{\text{k}}$$

$$k_{xy}^{\text{G}}=\left(\frac{1}{L_{\text{t}}^{2}L_{\text{s}}^{2}\sin{\text{θ}}_{\text{k}}^{3}}\right)\left(L_{\text{s}}^{2}\sin{\text{θ}}_{\text{hk}}\cos{\text{θ}}_{\text{hk}}\cos{\text{θ}}_{\text{k}}+L_{\text{s}}L_{\text{s}}\sin{\text{θ}}_{\text{h}}\cos{\text{θ}}_{\text{h}}\right)M_{\text{h}}-L_{\text{d}}^{2}\cos{\text{θ}}_{\text{d}}\sin{\text{θ}}_{\text{d}}\cos M_{\text{k}}$$

$$k_{yx}^{\text{G}}=k_{xy}^{\text{G}}$$

$$k_{yy}^{\text{G}}=\left(\frac{1}{L_{\text{t}}^{2}L_{\text{s}}^{2}\sin{\text{θ}}_{\text{k}}^{3}}\right)\left(L_{\text{s}}^{2}\cos{\text{θ}}_{\text{hk}}^{2}\cos{\text{θ}}_{\text{k}}+L_{\text{s}}L_{\text{t}}\cos{\text{θ}}_{\text{h}}^{2}\right)M_{\text{h}}-\left(L_{\text{d}}^{2}\cos{\text{θ}}_{\text{d}}^{2}\cos{\text{θ}}_{\text{k}}+L_{\text{s}}L_{\text{t}}\sin{\text{θ}}_{\text{k}}^{2}\right)M_{\text{k}}$$

### Muscular Damping (Tee et al. 2004)

$$B=\frac{0.1}{\sqrt{{\dot{p}}^{T}\dot{p}}}\left[\begin{array}{cc} k_{xx}^{\text{S}} & k_{xy}^{\text{S}}\\ k_{yx}^{\text{S}} & k_{yy}^{\text{S}} \end{array}\right]$$

### Forward Kinematics Transformation of Joint Moments to End-Point Forces

$$J=\left[\begin{array}{cc} -L_{\text{ha}}\sin{\text{θ}}_{\text{ha}} & -L_{\text{s}}\sin{\text{θ}}_{\text{hk}}\\ L_{\text{ha}}\cos{\text{θ}}_{\text{ha}} & L_{\text{s}}\cos{\text{θ}}_{\text{hk}} \end{array}\right]$$

$$T=J^{\text{T}}F$$

$$\left[\begin{array}{c} {\text{T}}_{\text{h}}\\ {\text{T}}_{\text{k}} \end{array}\right]=\left[\begin{array}{cc} -L_{\text{ha}}\sin{\text{θ}}_{\text{ha}} & L_{\text{ha}}\cos{\text{θ}}_{\text{ha}}\\ -L_{\text{s}}\sin{\text{θ}}_{\text{hk}} & L_{\text{s}}\cos{\text{θ}}_{\text{hk}} \end{array}\right]\left[\begin{array}{c} F_{x}\\ F_{y} \end{array}\right]$$

$$F=J^{-\text{T}}T$$

$$J^{-\text{T}}=\frac{1}{L_{\text{t}}L_{\text{s}}\sin{\text{θ}}_{\text{k}}}\left[\begin{array}{cc} L_{\text{s}}\cos{\text{θ}}_{\text{hk}} & -L_{\text{ha}}\cos{\text{θ}}_{\text{h}}-L_{\text{s}}\cos{\text{θ}}_{\text{hk}}\\ L_{\text{s}}\sin{\text{θ}}_{\text{hk}} & -L_{\text{ha}}\sin{\text{θ}}_{\text{h}}-L_{\text{s}}\sin{\text{θ}}_{\text{hk}} \end{array}\right]$$

$$\left[\begin{array}{c} F_{x}\\ F_{y} \end{array}\right]=\frac{1}{L_{\text{t}}L_{\text{s}}\sin{\text{θ}}_{\text{k}}}\left[\begin{array}{cc} L_{\text{s}}\cos{\text{θ}}_{\text{hk}} & -L_{\text{ha}}\cos{\text{θ}}_{\text{h}}-L_{\text{s}}\cos{\text{θ}}_{\text{hk}}\\ L_{\text{s}}\sin{\text{θ}}_{\text{hk}} & -L_{\text{ha}}\sin{\text{θ}}_{\text{h}}-L_{\text{s}}\sin{\text{θ}}_{\text{hk}} \end{array}\right]\left[\begin{array}{c} {\text{T}}_{\text{h}}\\ {\text{T}}_{\text{k}} \end{array}\right]$$

### Treadmill Contact Model

$$D=\sqrt{\left(p_{x}-p_{x,\text{Gnd}}\right)+\left(p_{y}-p_{y,\text{Gnd}}\right)}$$

$$Pen=R_{\text{GND}}/D$$

p^err=[px,Gnd+Pen(px−px,Gnd)py,Gnd+Pen(py−py,Gnd)]

## Abbreviations

*F_x_*: Horizontal force at ankle (end point)
F_*y*_: Vertical force at ankle (end point)
I_s_: Shank moment of inertia about center of gravity
I_t_: Thigh moment of inertia about center of gravity
*L*_ha_: Distance between hip and ankle
*L*_s_: Length of shank
*L*_t_: Length of thigh
*M*_h_: Hip torque due to hip muscular action
*M*_k_: Knee torque due to knee muscular action
*m*_s_: Mass of shank
*m*_t_: Mass of thigh
*p_x_*: Horizontal position of ankle
*p_y_*: Vertical position of ankle
*r*_s_: Distance to center of mass of shank from proximal end
*r*_t_: Distance to center of mass of thigh from proximal end
T_h_: Hip torque
T_k_: Knee torque
$\theta_{\text{h}},{\dot{\theta }}_{\text{h}},{\ddot{\theta }}_{\text{h}}$: Hip angle, angular velocity, and angular acceleration
$\theta_{\text{k}},{\dot{\theta }}_{\text{k}},{\ddot{\theta }}_{\text{k}}$: Knee angle, angular velocity, and angular acceleration
ρ_s_: Shank radius of gyration about center of gravity
ρ_t_: Thigh radius of gyration about center of gravity

## For Treadmill Model

*D*: Distance between the VP’s ankle and the treadmill
*Pen*: Ratio describing how far the leg penetrates the treadmill
*R*_GND_: Radius of treadmill surface from hip joint center
*R*_S_: Radius of virtual spheres; one was attached to VP’s ankle, and the other slid along a semicircular arc defining a modified virtual treadmill surface with radius *R*_GND_
Ψ_h_: Hip muscular spring stiffness

## Vectors and Matrices

B: Muscular damping matrix
F: Vector of end-point (ankle) forces
G: Matrix specifying joint torques due to gravity
I: Inertia matrix
J: Jacobian matrix
J^T^: Jacobian transpose matrix
J^−T^: Inverse Jacobian transpose matrix
***K***_SPR_: Muscular spring stiffness expressed at ankle end point
***K***_GEO_: Muscular geometric stiffness expressed at ankle end point
***p***_Gnd_: Position of the ankle at which it would contact the treadmill
***p̂***_err_: VP ankle position projected onto the surface of the treadmill sphere
T: Vector of joint torques
V: Matrix specifying joint torques from Coriolis and centrifugal forces

## Parameters (from Winter 1990, except height and weight)

*g*: Gravitational acceleration in Boston (9.82045 m/s^2^; from WolframAlpha)
ht: Height (1.70 m) (Olney and Richards 1996)
I_t_: $m_{\text{t}}{\text{ρ}}_{\text{t}}^{\text{2}}$
I_s_: $m_{\text{s}}{\text{ρ}}_{\text{s}}^{\text{2}}$
*L*_t_: 0.245ht
*L*_s_: 0.246ht
*m*_t_: 0.1wt
*m*_s_: 0.0456wt
*r*_t_: 0.433*L*_t_
*r*_s_: 0.433*L*_s_
wt: Weight (72.0 kg) (Olney and Richards 1996)
ρ_t_: 0.323*L*_t_
ρ_s_: 0.302*L*_s_

## Other Quantities

*p_x_*: *L*_t_sinθ_h_ + *L*_s_sinθ_hk_
*p_y_*: −*L*_t_cosθ_h_ − *L*_s_cosθ_hk_
*R*_GND_: 0.995(*L*_t_ + *L*_s_ + 14 mm)
θ_hk_: θ_h_ + θ_k_
θ_ha_: −atan(*p_x_*/*p_y_*)
